# Molecular Imaging in Nanomedical Research

**DOI:** 10.3390/ijms23084207

**Published:** 2022-04-11

**Authors:** Manuela Malatesta

**Affiliations:** Department of Neurosciences, Biomedicine and Movement Sciences, University of Verona, I-37134 Verona, Italy; manuela.malatesta@univr.it

For years, nanomedical research has represented a challenge and an opportunity in terms of imaging techniques. In fact, the development of safe and effective nanoconstructs designed for diagnostic or therapeutic purposes implies the knowledge of their fine structure and their interactions with the biological environment. The need to characterise morphologically newly synthesised nanoconstructs and localise them in living systems (from single cells to the whole organism) resulted in the application of the most varied imaging approaches. The biodistribution, targeting and clearance of nanomedical tools in the whole organism have been investigated using in vivo imaging techniques such as magnetic resonance imaging (MRI), optical imaging and positron emission tomography (PET), while the evaluation of the impact and dynamics of nanoparticulates in cells and tissues required the application of light and electron microscopy techniques [1,2,3,4]. In many studies, imaging techniques were combined to obtain more comprehensive knowledge of the biomedical potential of novel nanoconstructs, ranging from in vivo to in vitro approaches and from low- to high-resolution techniques. Interestingly, the most modern and refined technologies have often been associated with well-established molecular techniques in situ, such as immunohistochemistry, which became increasingly popular from the 1950s [5,6], or the even most classical histochemistry, dating back to the first half of the 19th century [7,8].

This Special Issue contains various examples of original applications of multimodal imaging to diverse nanomedical topics.

In an in vitro study, engineered polarity-sensitive fluorescent fatty acids derivatives were monitored for their distribution in cultured cells by fluorescence lifetime imaging, which provided a dynamic view of the fluidity and microviscosity of different cell membrane compartments [9]. Moreover, spectrum fluorescence imaging microscopy analysis was coupled with traditional histochemistry by using lipid- or lysosome-specific fluorescent dyes for their lifetime colocalisation with fatty acids derivatives, thus also making these probes suitable tools with which to observe lipophagy and related events.

In another in vitro study, various imaging techniques were applied to assess the suitability of ethosomes and transethosomes as potential delivery systems for vitamin D3 to different cell types [10]. Cryo-transmission electron microscopy (cryo-TEM) allowed the fine structural characterisation of the nanocarriers after vitamin D3 loading. Then, histochemical staining methods were used to track these lipid-based nanocarriers inside the cells at fluorescence microscopy and to check their effects on intracellular lipid deposits at bright field microscopy. Finally, TEM provided high-resolution information on the different intracellular fate of ethosomes and transethosomes with respect to their lipid composition.

As for in vivo studies, PET was used to map traumatic brain injury in rats by using two radioligands—[11C]peripheral benzodiazepine receptor (PBR) 28 and [18F]flumazenil—as indicators of neuroinflammation and neuronal activity and death [11]. The in vivo results were corroborated using an ex vivo immunohistochemical analysis of the distribution of a neuroinflammation marker in brain slices, thus creating promising perspectives for the utilisation of these radioligands in nanomedical therapies.

In vivo multimodal imaging and ex vivo investigations were performed to follow labelled lymphoma cells in mice [12]: 2-deoxy-2-[18F]fluoro-D-glucose ([18F]FDG) molecules were injected, and their preferential uptake by tumour cells was monitored in vivo using [18F]FDG PET and PET/MRI, and ex vivo using autoradiography, Cherenkov luminescence imaging and fibre-optic confocal endomicroscopy imaging. These data were also correlated with immunohistochemical analyses of organ sections using bright field microscopy.

Exosomes from cultured murine breast cancer cells were simultaneously radiolabelled, fluorescently labelled, and morphologically characterised using TEM to then be monitored with PET. Optical imaging both in living mice and in single explanted organs was carried out ex vivo [13]. The exosome distribution was confirmed at bright field and fluorescence microscopy using immunohistochemical analyses of tissue slices.

In addition to original experimental reports, this Special Issue provides two review articles that deal with advanced applications of molecular imaging techniques to nanomedicine.

Sier et al. [14] provided an overview of different cell-based tracers (living cells, cell-derived fragments, microorganisms, or nanoparticles) for targeted image-guided surgery, while Almeida et al. [15] reviewed the current radiolabelling strategies to track extracellular vesicles in vivo using radionuclide-based imaging techniques.

Taken together, the articles collected in this Special Issue affirm the vitality of the scientific research in the field of nanomedicine and the great contribution given by the original application of molecular imaging techniques to the development of innovative diagnostic and therapeutic strategies based on nanoconstructs.
